# Time-of-Day-Dependent Enhancement of Adult Neurogenesis in the Hippocampus

**DOI:** 10.1371/journal.pone.0003835

**Published:** 2008-12-02

**Authors:** So-ichi Tamai, Kamon Sanada, Yoshitaka Fukada

**Affiliations:** 1 Department of Biophysics and Biochemistry, Graduate School of Science, The University of Tokyo, Bunkyo-Ku, Tokyo, Japan; 2 Department of Developmental Neuroscience, Osaka University Graduate School of Medicine, Suita, Osaka, Japan; 3 PRESTO, Japan Science and Technology Agency, Kawaguchi, Saitama, Japan; James Cook University, Australia

## Abstract

**Background:**

Adult neurogenesis occurs in specific regions of the mammalian brain such as the dentate gyrus of the hippocampus. In the neurogenic region, neural progenitor cells continuously divide and give birth to new neurons. Although biological properties of neurons and glia in the hippocampus have been demonstrated to fluctuate depending on specific times of the day, it is unclear if neural progenitors and neurogenesis in the adult brain are temporally controlled within the day.

**Methodology/Principal Findings:**

Here we demonstrate that in the dentate gyrus of the adult mouse hippocampus, the number of M-phase cells shows a day/night variation throughout the day, with a significant increase during the nighttime. The M-phase cell number is constant throughout the day in the subventricular zone of the forebrain, another site of adult neurogenesis, indicating the daily rhythm of progenitor mitosis is region-specific. Importantly, the nighttime enhancement of hippocampal progenitor mitosis is accompanied by a nighttime increase of newborn neurons.

**Conclusions/Significance:**

These results indicate that neurogenesis in the adult hippocampus occurs in a time-of-day-dependent fashion, which may dictate daily modifications of dentate gyrus physiology.

## Introduction

Neurogenesis in the mammalian brain persists through adulthood mainly within the two neurogenic structures, the dentate gyrus of the hippocampus and the subventricular zone of the forebrain [1], [2]. In these areas neural progenitor cells continuously divide and give birth to new neurons [1]. Previous studies have demonstrated that behavioral and physiological stimuli such as learning [3], voluntary wheel running exercise [4], and environmental enrichment [5] enhance hippocampal neurogenesis. In addition, reproductive behaviors such as mating and pregnancy stimulate progenitor divisions and neurogenesis in the subventricular zone [6], [7]. Thus, proliferation of progenitors and neurogenesis in the adult brain are dynamic processes regulated by various internal and external stimuli specific to each neurogenic region.

In the hippocampus, neuronal properties such as excitability and connectivity are known to be modulated in a time-of-day-dependent manner. For example, features of long-term potentiation show day/night fluctuations in the hippocampus [8], and hippocampus-mediated learning is facilitated in daytime [9], [10]. In addition to the daily regulation of the hippocampal neurons, an apparent daily change in the number of S-phase cells has been reported in the hilus where proliferative glia reside [11]. Thus, glial proliferation seems to depend on the time of the day in the hilus. Although these observations suggest an intimate connection between the temporal information and hippocampal neurons/glia, it is unclear whether neural progenitor cells and neurogenesis in the dentate gyrus of the hippocampus are modulated in a time-of-day-dependent fashion. Given that the cell divisions in certain mammalian tissues (e.g., tongue epithelium, intestinal epithelium, and skin) associate with specific times of the day [12]–[14], we explored daily variations of neural progenitor divisions and neurogenesis in the adult mouse brain.

## Results

**Figure 1:**
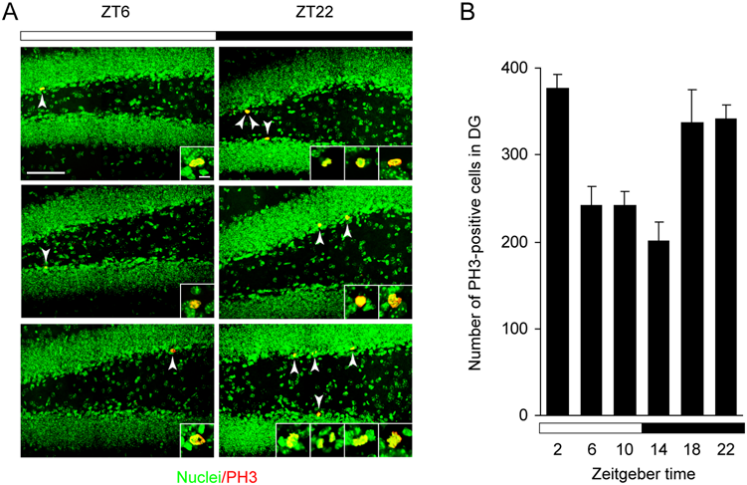
Daily variation of hippocampal cell proliferation. (A) Representative confocal images of PH3-positive cells (arrowheads) in the dentate gyrus (DG) at ZT 6 (three examples, left panels) and ZT 22 (three, right panels). Sections were labeled with PH3 antibody (red) and with DAPI (green). Scale bar, 50 µm. High-magnification images of each PH3-labeled cell are shown as insets (Scale bar, 5 µm). (B) Total numbers of PH3-positive cells per DG at various ZT indicated were counted and presented as mean±s.e.m. (*n* = 4 for each time point). There was a statistically significant effect of the time-of-day on the number of PH3-positive cells (*p* = 0.0001 by one-way ANOVA). The white and black bars represent light and dark periods of the day, respectively.

**Figure 2:**
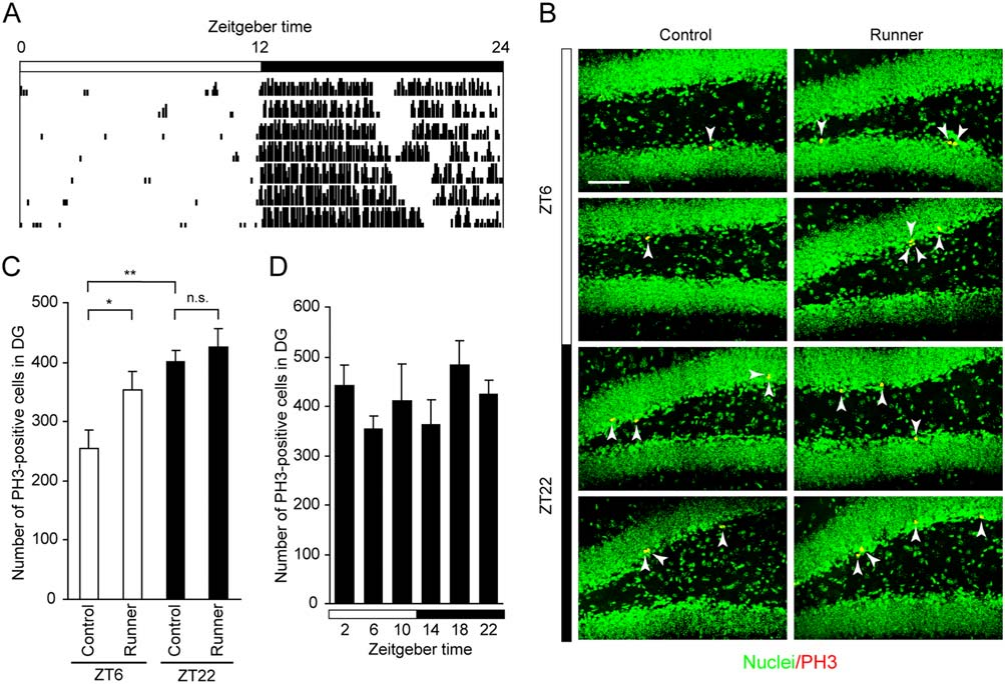
Effects of wheel running exercise on hippocampal cell divisions. Mice were divided into two groups: one group was housed in cages with a running wheel (runner) and the other group in cages with a locked wheel (control). (A) A representative actogram of the wheel running activity from a mouse reared under the light/dark cycles. (B) Confocal images of PH3-positive cells (arrowheads) in the hippocampus at ZT 6 and ZT 22. Sections were derived from runners and controls, and were labeled with PH3 antibody (red) and with DAPI (green). Scale bar, 50 µm. (C) Total numbers of PH3-labeled cells per DG in the control and runner groups at indicated ZT (mean±s.e.m., *n* = 6–8). *p<0.05, **p<0.01 by two-tailed student's *t*-test. n.s., not significant. (D) Total numbers of PH3-labeled cells per DG in the runner group at various ZT indicated (mean±s.e.m., *n* = 3–8 for each time point, *p* = 0.25 by one-way ANOVA).

Six-week-old male mice were housed individually under 12-hour light/12-hour dark cycles for 2 weeks and sacrificed at various Zeitgeber time (ZT; ZT 0 represents light on and ZT 12, light off in a 12-hour light/12-hour dark cycle). We then examined the number of dividing cells in the subgranular zone (SGZ) of the dentate gyrus by immunostaining with an antibody against phospho-histone H3 (PH3), a marker for M-phase cells. PH3-positive cells were mainly located in SGZ of the dentate gyrus where neurogenesis occurs (Figure 1A,B). The number of the PH3-positive cells in SGZ was significantly higher (*p*<0.0001) during the nighttime than during the daytime, showing a robust day/night variation of the timing of mitosis (Figure 1C). Progenitor proliferation in SGZ is known to be regulated by various stimuli such as voluntary wheel running exercise [4]. We therefore examined how the day/night variation of M-phase cells is affected by the running exercise that is mainly observed during the nighttime (Figure 2A). At ZT 6, a time when the number of M-phase cells is at the near trough in the standard housing (Figure 1), the PH3-labeled cell number was significantly elevated in SGZ of exercised animals (Figure 2B,C; *p*<0.05). By contrast, the voluntary exercise had no significant effect on the mitotic fraction at ZT 22 when the number of M-phase cells is high in the standard housing (Figure 2B,C), and exercised animals apparently showed constant levels of cell mitosis throughout the day/night cycle (Figure 2D). The time-specific effect of the voluntary exercise may be attributable to the time-restriction of the exercise or the time-of-day-dependent gating of the exercise-induced signals. Altogether, progenitor cells in SGZ show the daily change of M-phase cells that is modifiable by the nocturnal exercise.

**Figure 3:**
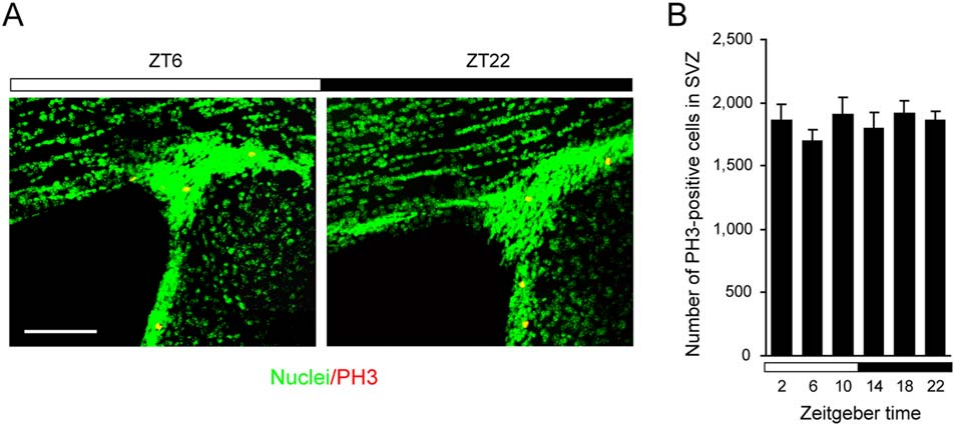
Temporal profile in the number of M-phase cells in the subventricular zone over the day. (A) Confocal images of PH3-positive cells in the subventricular zone at ZT 6 (left panel) and ZT 22 (right panel). Sections were labeled with the anti-PH3 antibody (red) and with DAPI (green). Scale bar, 50 µm. (B) Total numbers of PH3-positive cells per subventricular zone at various ZT indicated were counted and presented as mean±s.e.m. (*n* = 4 for each time point).

Noticeably, day/night variations of M-phase cells is region-specific, as the number of the PH3-positive cells in the subventricular zone of the forebrain was almost constant throughout the day (Figure 3A,B). We further characterized progenitor divisions in SGZ. The number of S-phase cells remained unchanged across the day, as determined by 30-min bromodeoxyuridine (BrdU) labeling (Figure S1), which is consistent with previous reports [11], [15]. These observations indicate that neural progenitor cells actively enter mitosis during the nighttime in SGZ.

**Figure 4:**
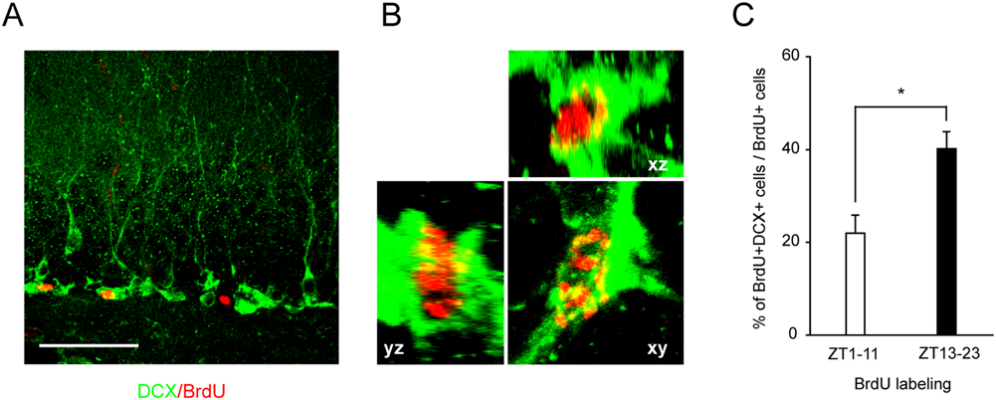
Nighttime stimulation of neurogenesis. (A) Confocal image of the subgranular zone of the DG labeled with antibodies against DCX (green) and BrdU (red). Scale bar, 50 µm. (B) Confocal images showing a DCX-BrdU double-labeled cell from orthogonal perspectives. (C) The percentage of DCX-BrdU double-labeled cells in the hippocampus of the mice that were injected with BrdU at ZT 1 (harvested at ZT 11) or at ZT 13 (harvested at ZT 23). Data are shown as mean±s.e.m. (*n* = 3 for each group). *p<0.05 by two-tailed student's *t*-test.

The increase in M-phase cells at nighttime raises the possibility that neurons are generated in higher proportions at night. To test this hypothesis, we analyzed the number of neuronal progeny generated through the cell divisions occurring during the daytime or nighttime. Mice were injected intraperitoneally with BrdU at ZT 1 for daytime analysis or ZT 13 for nighttime in order to label the dividing progenitors and they were sacrificed 10 hours later at ZT 11 or ZT 23, respectively. Under these experimental conditions, none of the BrdU-labeled cells were immunoreactive for NeuN, a marker of mature neurons (Figure S2). This short duration of labeling (10 hours) is probably insufficient for the labeled S-phase progenitors to differentiate into mature NeuN-expressing neurons. Therefore, we examined the immunoreactivity for doublecortin (DCX), a marker of immature neurons (Figure 4A,B). Among the cells that were BrdU-labeled during the daytime (BrdU-injected at ZT 1 and harvested at ZT 11), 22% were co-labeled with DCX. On the other hand, 40% of the nighttime-labeled cells (BrdU-injected at ZT 13 and harvested at ZT 23) were DCX-positive (Figure 4C). Furthermore, when progenitors were labeled with BrdU at different times of the day (ZT 5–ZT 15 and ZT 17–ZT 27), 23% (ZT 5–ZT 15) and 56% (ZT 17–ZT 27) of BrdU-labeled cells were DCX-positive (Figure S3). These observations indicate that new neurons are generated more frequently during the nighttime (*p*<0.05), and underscore the significance of the nighttime increase in progenitor divisions.

## Discussion

**Figure 5:**
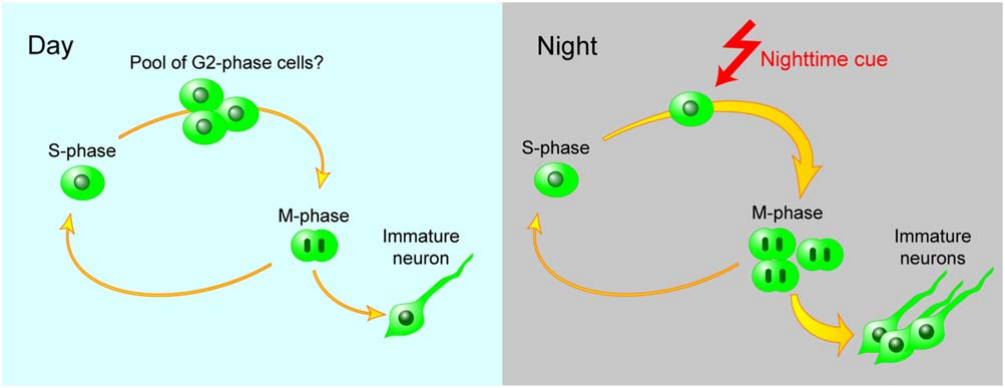
A model for the time-of-day-dependent enhancement of hippocampal neurogenesis. Hippocampal progenitors enter the cell cycle irrespective of time-of-day. The progression into M-phase is suppressed in the daytime, possibly by G2 arrest. At nighttime, the progenitors actively enter M-phase, thereby giving rise to more neuronal progeny.

In the present study, we found that M-phase cells show a clear day/night variation, with a significant increase during the night. On the other hand, the number of S-phase progenitors remains unchanged across the day, which is consistent with previous reports [11], [15]. Further, when progenitor divisions were characterized by immunostaining with an antibody against Thr161-phosphorylated form of cdc2, the immunoreactive cells showed a clear daily variation with its peak at night (Figure S4). Active cdc2 functions as an initiator of G2/M transition, and Thr161 phosphorylation is required for activation of cdc2 [16]. Therefore, we hypothesized that G2/M transition of progenitors is promoted during the night, which will result in the increase of M-phase cells. The increase in M-phase cells might also be attributable to an increase in length of M-phase at night, but it is less likely because mitotic delay/retardation of M-phase cells at night is not apparent, as judged from the distribution of mitotic cells in different phases (prophase, metaphase, ana/telophase) that did not change over the day (Figure S5). Based on these observations, we propose a model for the daily regulation of neural progenitor cells in the hippocampus (Figure 5). In this model, hippocampal progenitors enter the cell cycle as well as S-phase irrespective of time-of-day. The progression of these cells into M-phase is suppressed at daytime. At night, the progenitors actively enter M-phase, thereby giving rise to more neuronal progeny.

Interestingly, such a daily regulation of cell divisions is observed in certain types of tissues including tongue epithelium, intestinal epithelium, and skin [12]–[14], implying that the temporal regulation of progenitor divisions seems to be important for animals living under day/night cycles. Notably, a global increase in dentate gyrus activity is observed by enhanced neurogenesis [17], suggesting that newly generated neurons modify the properties of existing circuitry [18]. In this context, the daily variation of hippocampal neurogenesis may mediate a temporal modification of dentate gyrus physiology.

A variety of cellular events are controlled in a time-of-day-dependent fashion by virtue of endogenous circadian clocks that reside in various tissues [19], [20]. Noticeably, circadian clock-related genes such as *Cryptochromes* and *Periods* have been reported to have inhibitory roles for mitotic divisions in the liver [21], [22]. Together with the significant expression of clock-related genes in the hippocampus [23]–[26], it is expected that the daily regulation of progenitor divisions and neurogenesis may be dictated by the circadian clocks that localize in the hippocampus.

Abnormalities in circadian/daily rhythms have been suggested to underlie the development of bipolar disorder [27], [28]. Noticeably, bipolar disorder appears to be related to dysregulated hippocampal neurogenesis. In fact, an animal model of depression with stress exhibits reduced neurogenesis in the dentate gyrus [29], while lithium, a commonly prescribed mood stabilizer for bipolar disorder, enhances hippocampal neurogenesis [30]. These studies imply that properly regulated hippocampal neurogenesis may mediate mood stabilizing effect on bipolar disorder. Importantly, daily variations of progenitor cell divisions (Figure 1C) are more apparent in the ventral dentate gyrus (Figure S6), a region contributing to the regulation of emotion [18]. These observations together provide the idea that the three events—normal daily rhythms, mood stabilization, and proper neurogenesis—are interconnected. In this context, bipolar disorder induced by aberrant daily rhythms may in part rely on abnormalities in time-of-day-dependent hippocampal neurogenesis. Understanding the mechanisms underlying the temporal regulation of neurogenesis should provide insights not only into the etiology of bipolar disorder, but also the physiological role of hippocampal neurogenesis.

## Materials and Methods

### Housing conditions and wheel running exercise

Male mice (6-week-old) were housed individually under 12-hour light/12-hour dark cycles for two weeks, in order to synchronize the phase of their internal clocks to the light/dark cycles. All the cages were placed in light-tight cabinets where temperature (23±1°C) and humidity (55±10%) were kept constant. Animals had access to food and water *ad libitum*. Animal experiments were conducted in accordance with guidelines set by University of Tokyo and Osaka University.

For wheel running exercise, animals were kept in standard cages for 5 days prior to the wheel running exercise. Then they were divided into two groups, one in a cage with a running wheel (runner) while the other with a locked wheel (control), and both groups were held for 9 days. Revolutions of the running wheel were registered on a computer, and the data were plotted as an actogram. In the actogram (Figure 2A), the horizontal line represents one day, and the height of black vertical bars plotted side-by-side represent the relative number of wheel revolutions within a period of 5 minutes.

### Immunohistochemistry

Brains isolated at various ZT were cut into coronal slices (4-mm thickness) which include the entire hippocampus or subventricular zone. The brain slices were fixed in 4% paraformaldehyde/PBS (10 mM Na-phosphate and 140 mM NaCl, pH 7.4) overnight at 4°C. Serial sections were made using a vibratome. Brain sections (40 µm) were incubated with a blocking solution for 1 hour at room temperature and then incubated with primary antibodies for 24–48 hours at 4°C. Cell nuclei were stained with DAPI.

Primary antibodies used were anti-phospho-histone H3 polyclonal antibody (1∶500; Upstate), anti-BrdU mouse monoclonal antibody (1∶500; Dako), anti-BrdU rat monoclonal antibody (1∶200; Funakoshi), anti-DCX rabbit polyclonal antibody (1∶500; Abcam), anti-NeuN mouse monoclonal antibody (1∶100; Chemicon), and phospho-cdc2 (Thr161) polyclonal antibody (1∶200; Cell Signaling). Blocking solutions used were PBS containing 3% BSA and 0.2% Triton X-100 (for PH3 staining), PBS containing 5% fetal bovine serum and 0.2% Triton X-100 (for BrdU/DCX staining), TBS (25 mM Tris-HCl [pH 7.4] and 140 mM NaCl) containing 5% fetal bovine serum and 0.3% Triton X-100 (for BrdU/NeuN staining), and TBS containing 3% BSA and 0.2% Triton X-100 (for phospho-cdc2 staining).

For BrdU labeling, animals intraperitoneally received a single dose of BrdU (100 µg/g body weight for 30 minutes labeling, and 50 µg/g for 10 hours labeling) at a concentration of 5 mg/ml. The brain sections were pretreated with 4 N HCl for 15 minutes at room temperature (for BrdU and DCX double-staining) or 1 N HCl for 1 hour at 37°C (for BrdU and NeuN double-staining) in order to unmask the antigens, before immunohistochemistry.

For phospho-cdc2 staining, the sections were pretreated with HistoVT One (Nacalai tesque) for 20 minutes at 70°C in order to unmask the antigen before immunohistochemistry.

### Quantification

To assess the number of PH3-positive cells and BrdU-positive cells, every third section of the entire hippocampus and all sections of the subventricular zone were analyzed. For phospho-cdc2-positive cells, every sixth section of the entire hippocampus was analyzed. The number of the immunopositive cells in the hippocampus was multiplied by 3 (or 6 for phospho-cdc2 staining) to provide an estimate for the total number of the positive cells in the dentate gyrus. To determine the cell fate, more than 50 BrdU-positive cells per animal were analyzed for coexpression of BrdU and DCX, and ratios of double-labeled cells were determined. To determine the mitotic stages of M-phase cells, more than 25 PH3-positive cells per animal were analyzed for their chromosomal shapes and percentages of the cells in each stage were determined.

## Supporting Information

Figure S1 Temporal profile in the number of S-phase cells in the hippocampus across the day. (A) Confocal images of BrdU-positive cells (green) in the dentate gyrus (DG) at ZT 6 (left panel) and ZT 22 (right panel). The granule cell layers are outlined with dashed lines. Scale bar, 50 µm. (B) Total numbers of BrdU-labeled cells per DG were counted at various ZT indicated and presented as mean±range (n = 2 for each time point).
(0.35 MB TIF)

Figure S2 Confocal images of the subgranular zone of DG labeled with antibodies against NeuN (green) and BrdU (red). Scale bar, 50 µ.
(0.43 MB TIF)

Figure S3 Nighttime stimulation of neurogenesis. The percentage of DCX-BrdU double-labeled cells in the hippocampus of the mice that were injected with BrdU at ZT 5 (harvested at ZT 15) or at ZT 17 (harvested at ZT 27). Data are shown as mean±s.e.m. (n = 3 for each group). **p<0.01 by two-tailed student's t-test.
(0.13 MB TIF)

Figure S4 Daily variation of phospho-cdc2-positive cells. Sections were immunostained with the antibody against Thr161-phosphorylated form of cdc2. (A) Phospho-cdc2-positive cells were mainly located in SGZ of the DG, and majority (>75%) of the cells was prior to or in early stage of mitosis, as assessed by the shapes of their chromosomes. Scale bar, 10 µm. (B) Total numbers of phospho-cdc2-positive cells per DG at various ZT indicated were counted and presented as mean±s.e.m. (n = 3 for each time point). There was a statistically significant effect of the time-of-day on the number of phospho-cdc2-positive cells (p<0.01 by one-way ANOVA).
(0.42 MB TIF)

Figure S5 Mitotic stages of PH3-positive cells. (A) Representative confocal images of the PH3-positive cells in ana/telophase (top), metaphase (middle), and prophase (bottom). Scale bar, 5 µm. (B) Distribution of PH3-positive mitotic cells in different phases at various ZT indicated (mean±s.e.m, n = 4 for each time point).
(0.46 MB TIF)

Figure S6 Daily variation of hippocampal cell proliferation. (A) Total numbers of PH3-positive cells per dorsal DG at various ZT indicated are presented as mean±s.e.m. (n = 4 for each time point). (B) Total numbers of PH3-positive cells per ventral DG. (C) Total numbers of PH3-positive cells per DG (the same figure as Figure 1C).
(0.24 MB TIF)

## References

[1] Ming GL, Song H (2005). Adult neurogenesis in the mammalian central nervous system.. Annu Rev Neurosci.

[2] Alvarez-Buylla A, Garcia-Verdugo JM (2002). Neurogenesis in adult subventricular zone.. J Neurosci.

[3] Gould E, Beylin A, Tanapat P, Reeves A, Shors TJ (1999). Learning enhances adult neurogenesis in the hippocampal formation.. Nat Neurosci.

[4] van Praag H, Kempermann G, Gage FH (1999). Running increases cell proliferation and neurogenesis in the adult mouse dentate gyrus.. Nat Neurosci.

[5] Kempermann G, Kuhn HG, Gage FH (1997). More hippocampal neurons in adult mice living in an enriched environment.. Nature.

[6] Shingo T, Gregg C, Enwere E, Fujikawa H, Hassam R (2003). Pregnancy-stimulated neurogenesis in the adult female forebrain mediated by prolactin.. Science.

[7] Mak GK, Enwere EK, Gregg C, Pakarainen T, Poutanen M (2007). Male pheromone-stimulated neurogenesis in the adult female brain: possible role in mating behavior.. Nat Neurosci.

[8] Chaudhury D, Wang LM, Colwell CS (2005). Circadian regulation of hippocampal long-term potentiation.. J Biol Rhythms.

[9] Chaudhury D, Colwell CS (2002). Circadian modulation of learning and memory in fear-conditioned mice.. Behav Brain Res.

[10] Valentinuzzi VS, Menna-Barreto L, Xavier GF (2004). Effect of circadian phase on performance of rats in the Morris water maze task.. J Biol Rhythms.

[11] Kochman LJ, Weber ET, Fornal CA, Jacobs BL (2006). Circadian variation in mouse hippocampal cell proliferation.. Neurosci Lett.

[12] Scheving LE, Burns ER, Pauly JE, Tsai TH (1978). Circadian variation in cell division of the mouse alimentary tract, bone marrow and corneal epithelium.. Anat Rec.

[13] Bjarnason GA, Jordan R (2002). Rhythms in human gastrointestinal mucosa and skin.. Chronobiol Int.

[14] Bjarnason GA, Jordan RC, Sothern RB (1999). Circadian variation in the expression of cell-cycle proteins in human oral epithelium.. Am J Pathol.

[15] Holmes MM, Galea LA, Mistlberger RE, Kempermann G (2004). Adult hippocampal neurogenesis and voluntary running activity: circadian and dose-dependent effects.. J Neurosci Res.

[16] Morgan DO (1995). Principles of CDK regulation.. Nature.

[17] Airan RD, Meltzer LA, Roy M, Gong Y, Chen H (2007). High-speed imaging reveals neurophysiological links to behavior in an animal model of depression.. Science.

[18] Sahay A, Hen R (2007). Adult hippocampal neurogenesis in depression.. Nat Neurosci.

[19] Reppert SM, Weaver DR (2002). Coordination of circadian timing in mammals.. Nature.

[20] Hastings MH, Reddy AB, Maywood ES (2003). A clockwork web: circadian timing in brain and periphery, in health and disease.. Nat Rev Neurosci.

[21] Matsuo T, Yamaguchi S, Mitsui S, Emi A, Shimoda F (2003). Control mechanism of the circadian clock for timing of cell division in vivo.. Science.

[22] Fu L, Pelicano H, Liu J, Huang P, Lee C (2002). The circadian gene Period2 plays an important role in tumor suppression and DNA damage response in vivo.. Cell.

[23] Antoch MP, Song EJ, Chang AM, Vitaterna MH, Zhao Y (1997). Functional identification of the mouse circadian Clock gene by transgenic BAC rescue.. Cell.

[24] Lamont EW, Robinson B, Stewart J, Amir S (2005). The central and basolateral nuclei of the amygdala exhibit opposite diurnal rhythms of expression of the clock protein Period2.. Proc Natl Acad Sci U S A.

[25] Guilding C, Piggins HD (2007). Challenging the omnipotence of the suprachiasmatic timekeeper: are circadian oscillators present throughout the mammalian brain?. Eur J Neurosci.

[26] Feillet CA, Mendoza J, Albrecht U, Pevet P, Challet E (2008). Forebrain oscillators ticking with different clock hands.. Mol Cell Neurosci.

[27] McClung CA (2007). Circadian genes, rhythms and the biology of mood disorders.. Pharmacol Ther.

[28] Roybal K, Theobold D, Graham A, DiNieri JA, Russo SJ (2007). Mania-like behavior induced by disruption of CLOCK.. Proc Natl Acad Sci U S A.

[29] Gould E, Tanapat P, McEwen BS, Flugge G, Fuchs E (1998). Proliferation of granule cell precursors in the dentate gyrus of adult monkeys is diminished by stress.. Proc Natl Acad Sci U S A.

[30] Chen G, Rajkowska G, Du F, Seraji-Bozorgzad N, Manji HK (2000). Enhancement of hippocampal neurogenesis by lithium.. J Neurochem.

