# Supplementary figures and images for: Time-of-Day-Dependent Enhancement of Adult Neurogenesis in the Hippocampus
Source: PLoS One. 2008 Dec 2;3(12):e3835. doi: 10.1371/journal.pone.0003835 (PMC2585014; doi:10.1371/journal.pone.0003835)

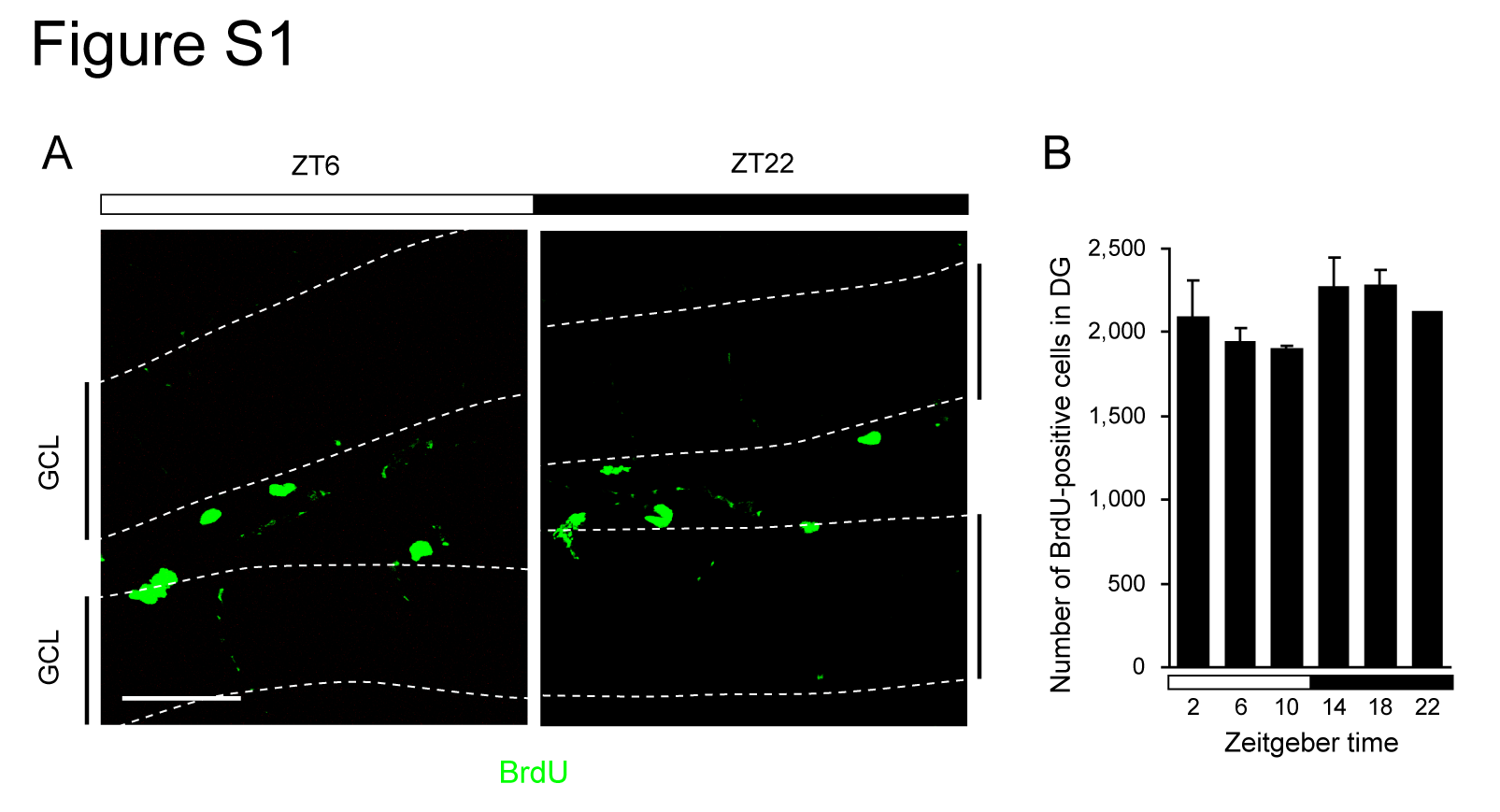

Supplement: Figure S1 — Temporal profile in the number of S-phase cells in the hippocampus across the day. (A) Confocal images of BrdU-positive cells (green) in the dentate gyrus (DG) at ZT 6 (left panel) and ZT 22 (right panel). The granule cell layers are outlined with dashed lines. Scale bar, 50 µm. (B) Total numbers of BrdU-labeled cells per DG were counted at various ZT indicated and presented as mean±range (n = 2 for each time point). (0.35 MB TIF) [file pone.0003835.s001.tif]

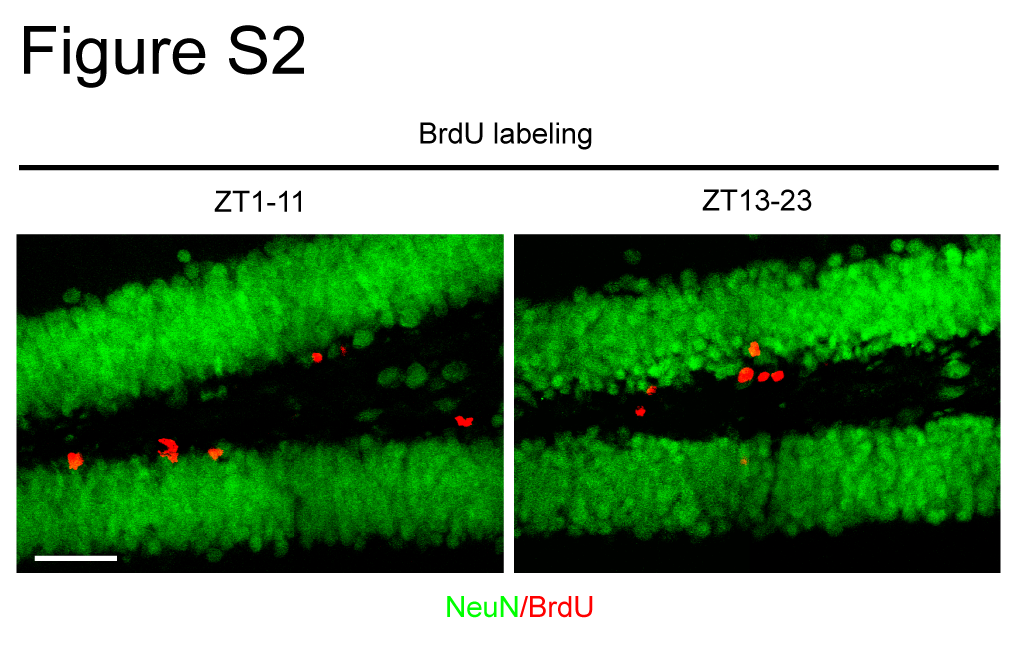

Supplement: Figure S2 — Confocal images of the subgranular zone of DG labeled with antibodies against NeuN (green) and BrdU (red). Scale bar, 50 µ. (0.43 MB TIF) [file pone.0003835.s002.tif]

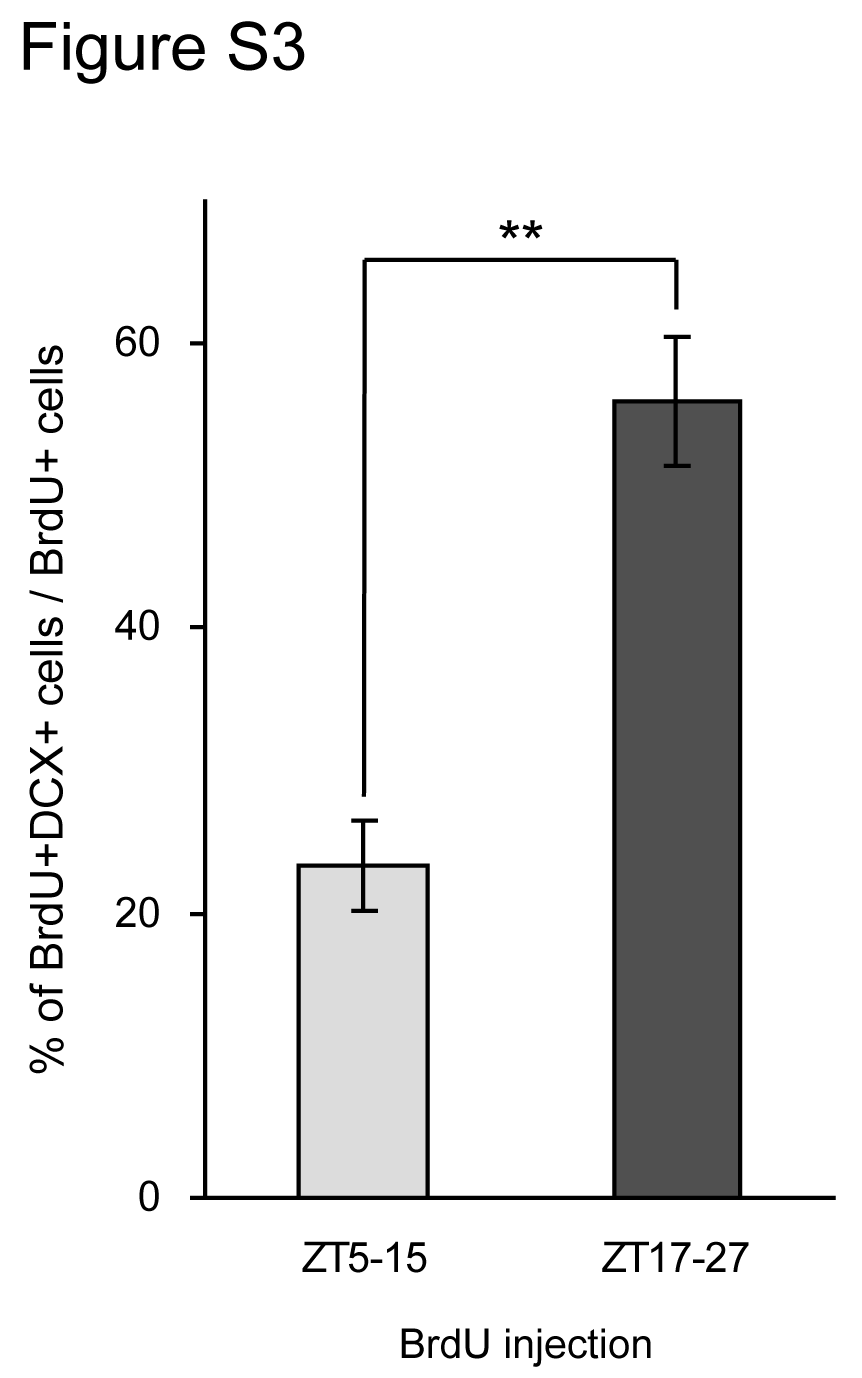

Supplement: Figure S3 — Nighttime stimulation of neurogenesis. The percentage of DCX-BrdU double-labeled cells in the hippocampus of the mice that were injected with BrdU at ZT 5 (harvested at ZT 15) or at ZT 17 (harvested at ZT 27). Data are shown as mean±s.e.m. (n = 3 for each group). **p<0.01 by two-tailed student's t-test. (0.13 MB TIF) [file pone.0003835.s003.tif]

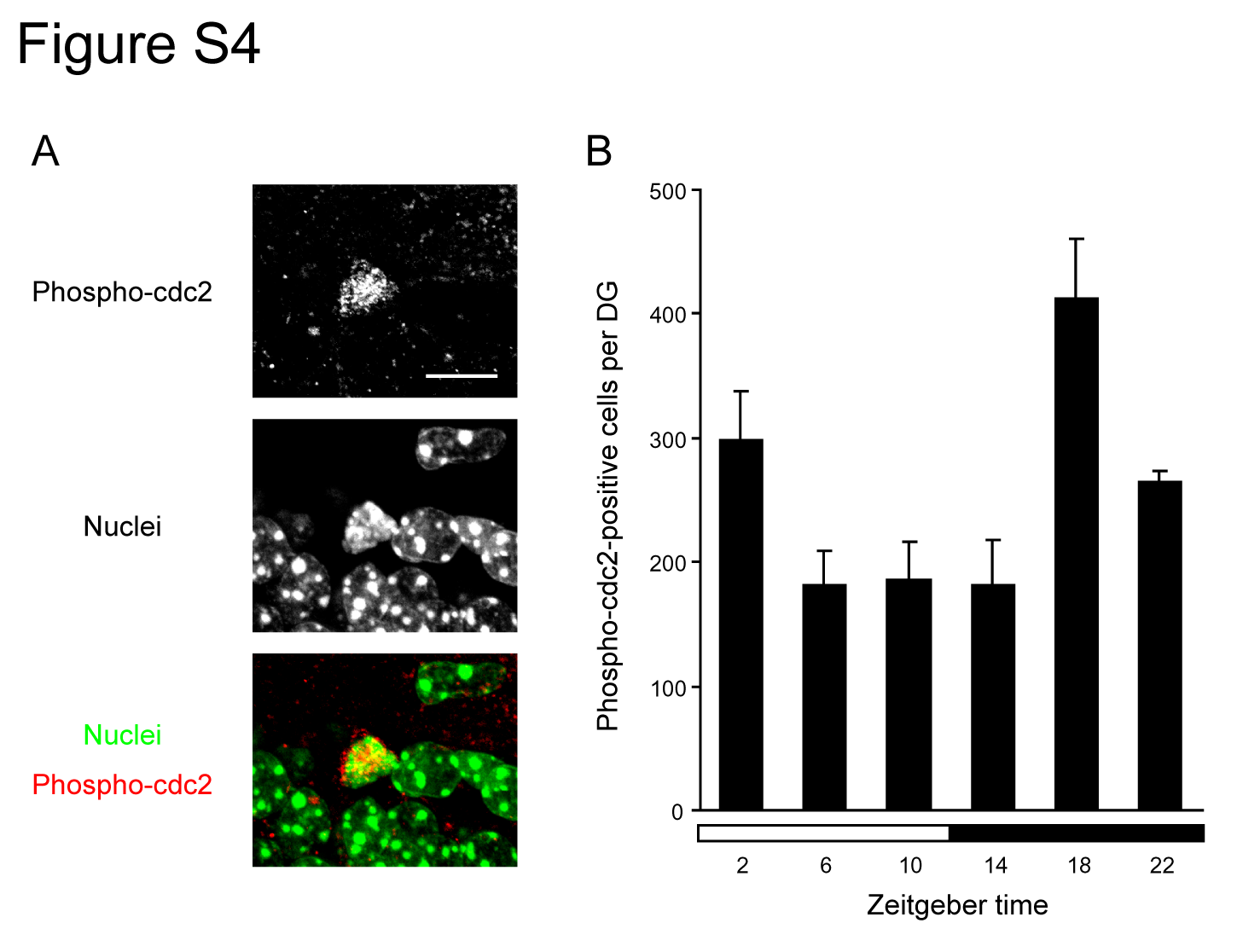

Supplement: Figure S4 — Daily variation of phospho-cdc2-positive cells. Sections were immunostained with the antibody against Thr161-phosphorylated form of cdc2. (A) Phospho-cdc2-positive cells were mainly located in SGZ of the DG, and majority (>75%) of the cells was prior to or in early stage of mitosis, as assessed by the shapes of their chromosomes. Scale bar, 10 µm. (B) Total numbers of phospho-cdc2-positive cells per DG at various ZT indicated were counted and presented as mean±s.e.m. (n = 3 for each time point). There was a statistically significant effect of the time-of-day on the number of phospho-cdc2-positive cells (p<0.01 by one-way ANOVA). (0.42 MB TIF) [file pone.0003835.s004.tif]

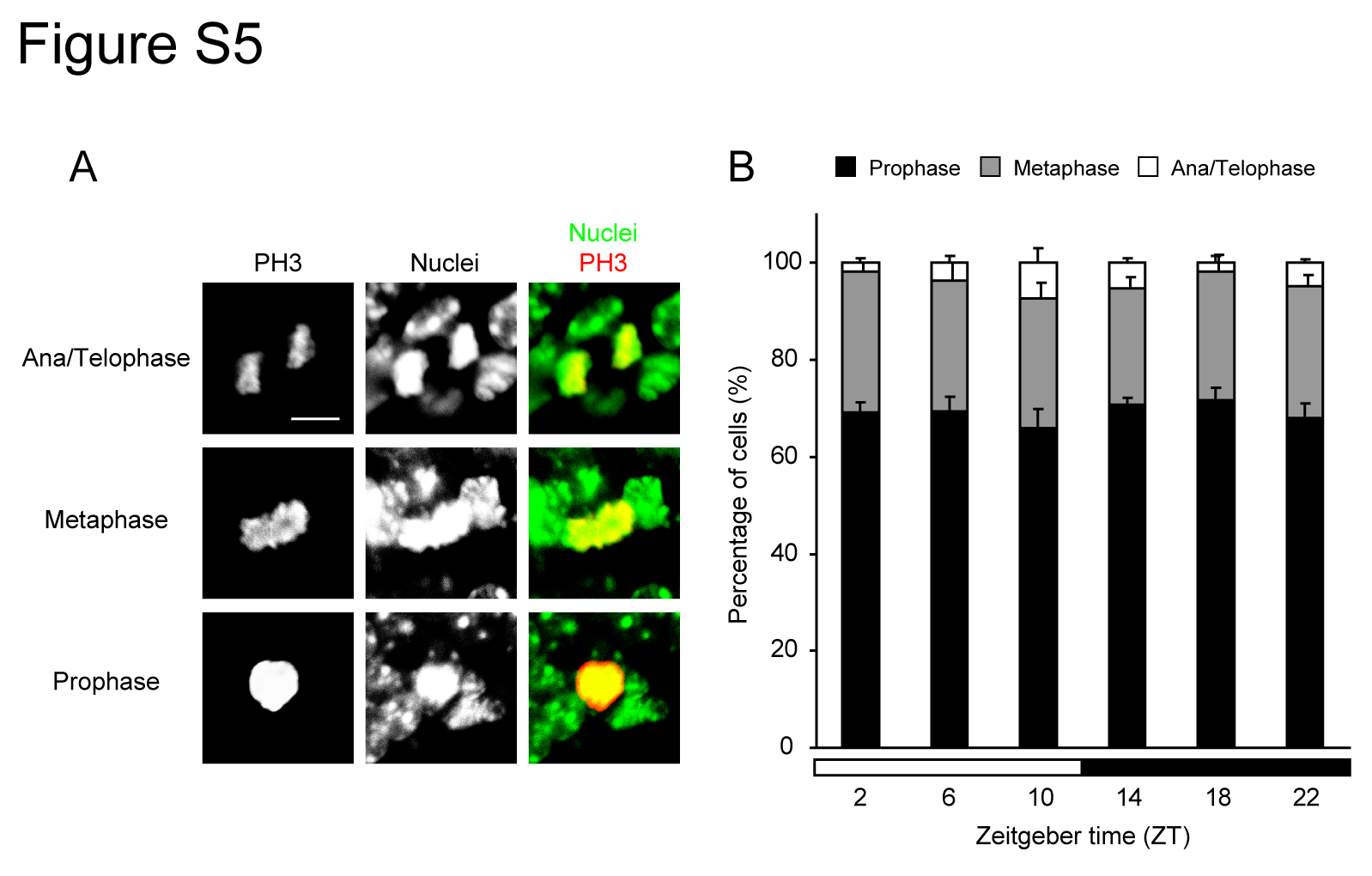

Supplement: Figure S5 — Mitotic stages of PH3-positive cells. (A) Representative confocal images of the PH3-positive cells in ana/telophase (top), metaphase (middle), and prophase (bottom). Scale bar, 5 µm. (B) Distribution of PH3-positive mitotic cells in different phases at various ZT indicated (mean±s.e.m, n = 4 for each time point). (0.46 MB TIF) [file pone.0003835.s005.tif]

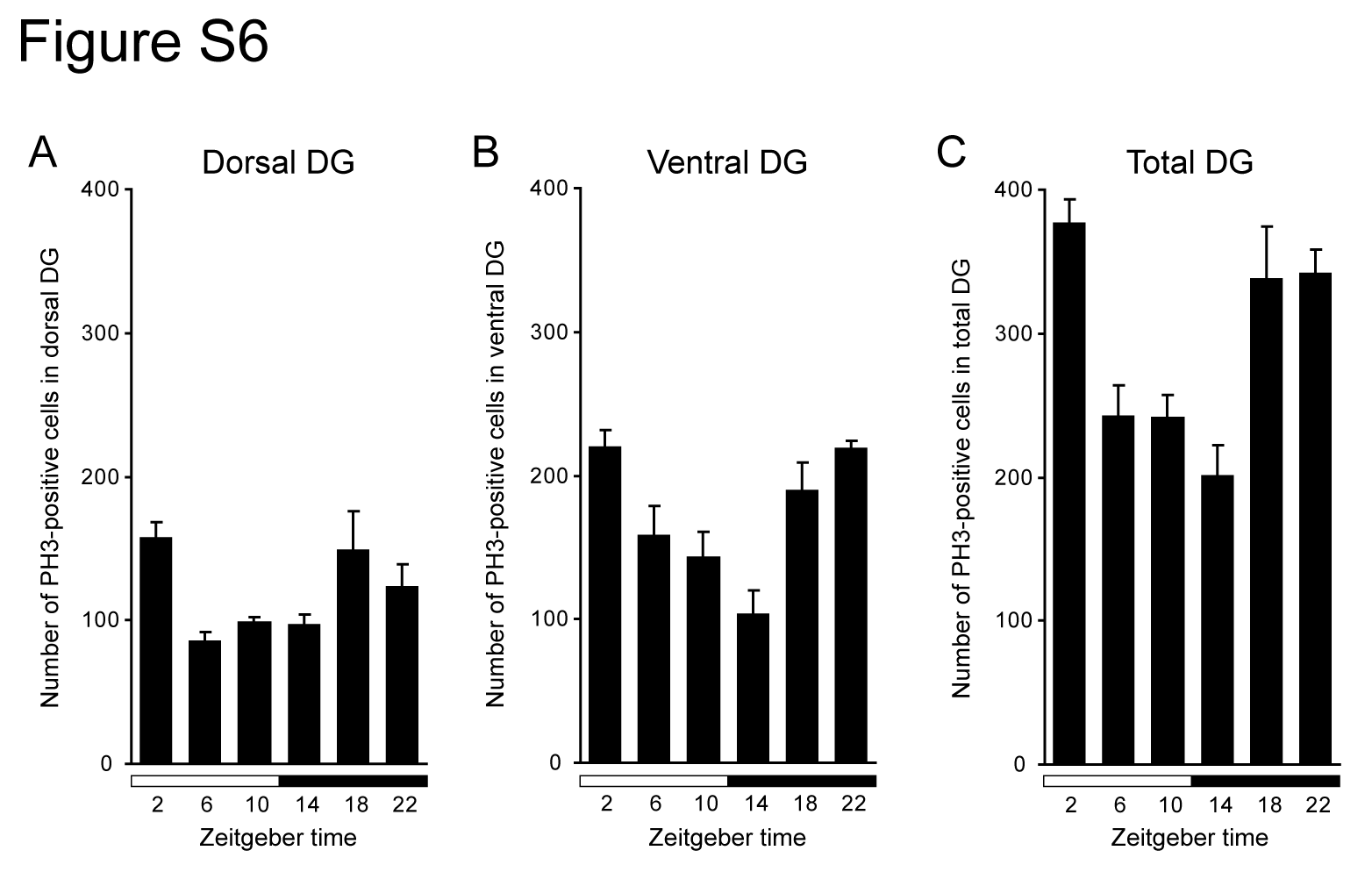

Supplement: Figure S6 — Daily variation of hippocampal cell proliferation. (A) Total numbers of PH3-positive cells per dorsal DG at various ZT indicated are presented as mean±s.e.m. (n = 4 for each time point). (B) Total numbers of PH3-positive cells per ventral DG. (C) Total numbers of PH3-positive cells per DG (the same figure as Figure 1C). (0.24 MB TIF) [file pone.0003835.s006.tif]
